# CAST-HSROC: A Web Application for Calculating the Summary Points of Diagnostic Test Accuracy From the Hierarchical Summary Receiver Operating Characteristic Model

**DOI:** 10.7759/cureus.13257

**Published:** 2021-02-10

**Authors:** Masahiro Banno, Yasushi Tsujimoto, Yan Luo, Chisato Miyakoshi, Yuki Kataoka

**Affiliations:** 1 Department of Psychiatry, Seichiryo Hospital, Nagoya, JPN; 2 Department of Psychiatry, Nagoya University Graduate School of Medicine, Nagoya, JPN; 3 Department of Systematic Reviewers, Systematic Review Workshop Peer Support Group (SRWS-PSG), Osaka, JPN; 4 Department of Healthcare Epidemiology, Graduate School of Medicine and Public Health, Kyoto University, Kyoto, JPN; 5 Department of Nephrology and Dialysis, Kyoritsu Hospital, Kawanishi, JPN; 6 Department of Health Promotion and Human Behavior, School of Public Health in the Graduate School of Medicine, Kyoto University, Kyoto, JPN; 7 Department of Research Support, Center for Clinical Research and Innovation, Kobe City Medical Center General Hospital, Kobe, JPN; 8 Hospital Care Research Unit, Hyogo Prefectural Amagasaki General Medical Center, Amagasaki, JPN; 9 Department of Respiratory Medicine, Hyogo Prefectural Amagasaki General Medical Center, Amagasaki, JPN

**Keywords:** grade, diagnostic test accuracy, systematic reviews, clinical practice guideline

## Abstract

Background: Researchers have been advised to report the point estimate of either sensitivity or specificity and its 95% credible interval (CrI) for a fixed specificity or sensitivity value in the summary of findings (SoF) table for diagnostic test accuracy (DTA) when they use the hierarchical summary receiver operating characteristic (HSROC) model. However, there is no other tool that easily calculates the statistics.

Results: We developed the calculator for the summary points from the HSROC model (CAST-HSROC), a web application for calculating the statistics easily. The existing graphical user interface software such as Review Manager and MetaDTA cannot calculate the statistics. Users should check whether convergence is reached before interpreting the results.

Conclusions: CAST-HSROC can easily calculate the point estimate of either sensitivity or specificity and its 95% CrI for a fixed specificity or sensitivity value on the HSROC model. The application can help to create an SoF table for DTA when systematic reviewers or guideline developers estimate the certainty of evidence on the HSROC model.

## Introduction

The number of systematic reviews (SR) has been increasingly used to assess diagnostic test accuracy (DTA) [1]. Two models are routinely used for the meta-analysis of DTA studies, namely, the bivariate and hierarchical summary receiver operating characteristic (HSROC) models [2]. The bivariate model produces summary estimates of sensitivity and specificity, whereas the HSROC model produces a summary receiver operating characteristic (ROC) curve. Their use depends on whether the diagnostic thresholds for the target condition used in the primary studies are similar. If the thresholds vary across the primary studies, the pooled estimates of sensitivity and specificity based on the bivariate model will be uninterpretable. In such a case (the HSROC model), those estimations are unrestricted by the threshold and should be appropriate. Recently, the Grading of Recommendations Assessment, Development and Evaluation (GRADE) Working Group has published new guidelines for estimating the certainty of evidence to help researchers in performing SR for DTA [3,4]. However, these guidelines were based on summary estimates of sensitivity and specificity and did not describe how the certainty of evidence can be estimated in GRADE for DTA when using the HSROC model. In contrast, the Cochrane Handbook suggests that researchers should report the point estimate of either sensitivity or specificity and its 95% credible interval (CrI) for a fixed specificity or sensitivity value in the summary of findings (SoF) table for DTA when they use the HSROC model, which is a statistical model based on latent-scale logistic regression. It considers the variabilities both within and between studies (for example, different cut-off values used in different primary studies) [2]. However, a difficult calculation based on the natural logarithm of the diagnostic odds ratio (log DOR) is required to obtain these values [2]. The calculation was performed using a complex equation. In the equation, Λ is the estimated average location parameter, and β is a scale parameter.

We need a new graphical user interface (GUI) software to easily calculate the point estimate of either sensitivity or specificity and its 95% CrI for a fixed specificity or sensitivity value because the existing software, namely Review Manager, MetaDTA, SAS, and R, cannot calculate the statistics in the GUI. Researchers in low-income countries and citizen scientists need the software for HSROC models with GUI environments because they might lack research funding and cannot consult statisticians who can implement HSROC models in a character user interface (CUI) environment. Moreover, access to a statistician is more restricted than access to statistical software in many settings [5]. Our objective was to develop a freely available web-based software that permits users to input their own data and generate the point estimate of either sensitivity or specificity and its 95% CrI for a fixed specificity or sensitivity value of the HSROC model in a DTA study.

## Materials and methods

**Figure 1:**
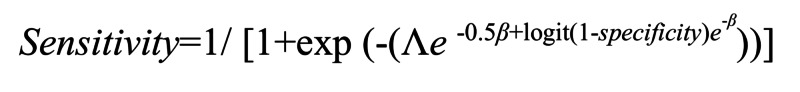
Equation of the HSROC model Λ is the estimated average location parameter, β is a scale parameter. HSROC: hierarchical summary receiver operating characteristic [2].

We developed the calculator for the summary points from the HSROC model (CAST-HSROC), a web calculator, to easily calculate the point estimate of either sensitivity or specificity and its 95% CrI for a fixed specificity or sensitivity value in the HSROC model [6]. We released the web application in March 2020. Thanks to this software, we no longer need to directly substitute values into complex mathematical formulas for calculations (Figure 1).

Software

We used the software R and its packages Shiny and RStan to develop the application [7-9]. Shiny is a package that permits users to develop web applications without executing programming using web development languages [8]. RStan is a package with executing programming by Stan [9]. Stan is a probabilistic programming language that performs Bayesian statistical inference via Markov Chain Monte Carlo [9]. CAST-HSROC works on the Shiny application server, which any user can use with a web browser, without any statistical software. The web application is available at https://youkiti.shinyapps.io/CAST-HSROC/ [6].

The inference for the Stan model is HSROC.

Setting of Markov chain Monte Carlo methods (MCMC) is as follows: four chains, each with iter=1000; warmup=500; thin=1; post-warmup draws per chain=500, total post-warmup draws=2000. Here, “chain” denotes the number of Markov chains; “iter” is the number of iterations for each chain; “warmup” is the number of warmup iterations per chain, and “thin” is the period for saving samples [9].

Data import

Users should upload their datasets before calculation. They can update it after clicking the tab 'Upload Data'.

The file should be in the formats that use delimiter-separated values (DSV), that is, to store two-dimensional arrays of data by separating the values in each row with specific delimiter characters. The supported delimiters are comma (,), semicolon (;), tab ( ), and space ( ). Please ensure you select the corresponding file delimiter in the left panel. We recommend uploading a comma-separated values (CSV) file.

The dataset should have five columns. Column 1 should be named as 'study_name', referring to the study ID, which can be numeric or characters. Each study contains fourfold (2 x 2) table information. Column 2 should be named as 'TP', and it includes the number of true positive patients (diseased patients with positive test results). Column 3 should be named as 'FN', and it includes the number of false-negative patients (diseased patients with negative test results). Column 4 should be named as 'FP', and it includes the number of false-positive patients (patients who are not diseased but have positive test results). Column 5 should be named as 'TN', and it includes the number of true-negative patients (patients who are not diseased and have negative test results).

If users upload the dataset successfully, they can visualize their data by clicking the tab 'Data Confirmation'.

CAST-HSROC includes the default example dataset “example data_set.csv” to help users understand its function. Users can also download it. We present the example dataset in Table 1.

**Table 1:** Example dataset TP: true positive, FN: false negative, FP: false positive, TN: true negative.

Study_name	TP	FN	FP	TN
1	90	10	20	80
2	40	10	5	45
3	60	40	10	90
4	200	50	100	200
5	10	5	5	15
6	80	20	10	90
7	60	40	30	70
8	8	2	1	9
9	250	50	100	200

Input parameter

Users need to input the parameter, sensitivity or specificity, after clicking the tab 'Results'. The sensitivity or specificity parameter can be selected based on the clinical experience or previous studies. For example, it can be the median value of the sensitivity or specificity reported by the studies included in the meta-analysis.

Functions

**Figure 2:**
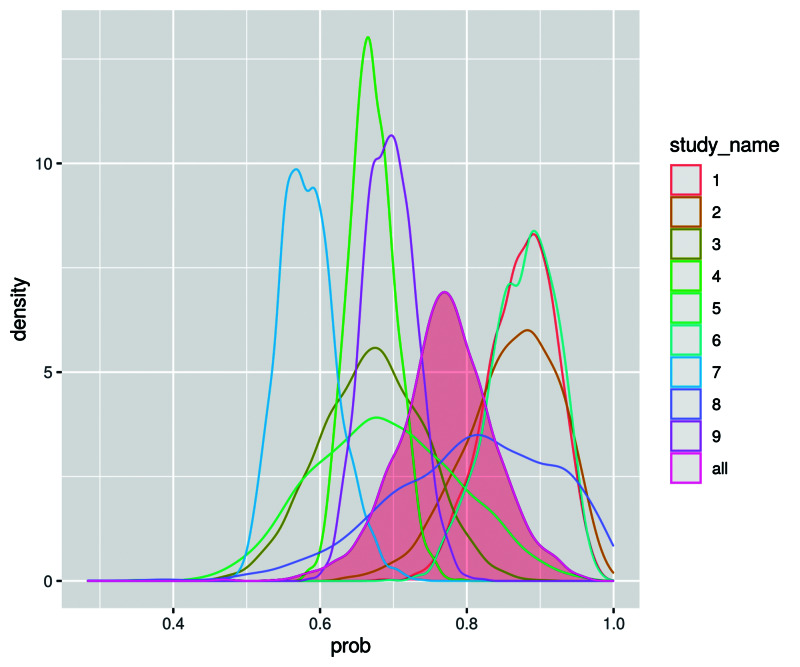
Example of the probability density plot prob: probability. “study_name” is the study identification number.

In reality, CAST-HSROC directly fits the HSROC model developed by Rutter and Gatsonis [10,11]. For prior distributions, we used non-informative uniform distributions with appropriate upper or lower limits. Appropriate limits that we used are “Uniform(0,+inf)” for standard deviations (SDs) and “Uniform(0,1)” for probabilities. CAST-HSROC calculates the estimated sensitivity or specificity and its 95% Crl after input from users. The application also shows a probability density plot of sensitivity or specificity. The distribution of the estimated variable is colored. Users can download the probability density plot as a PNG file. Figure 2 is an example of the probability density plot of sensitivity if specificity is 0.8.

**Figure 3:**
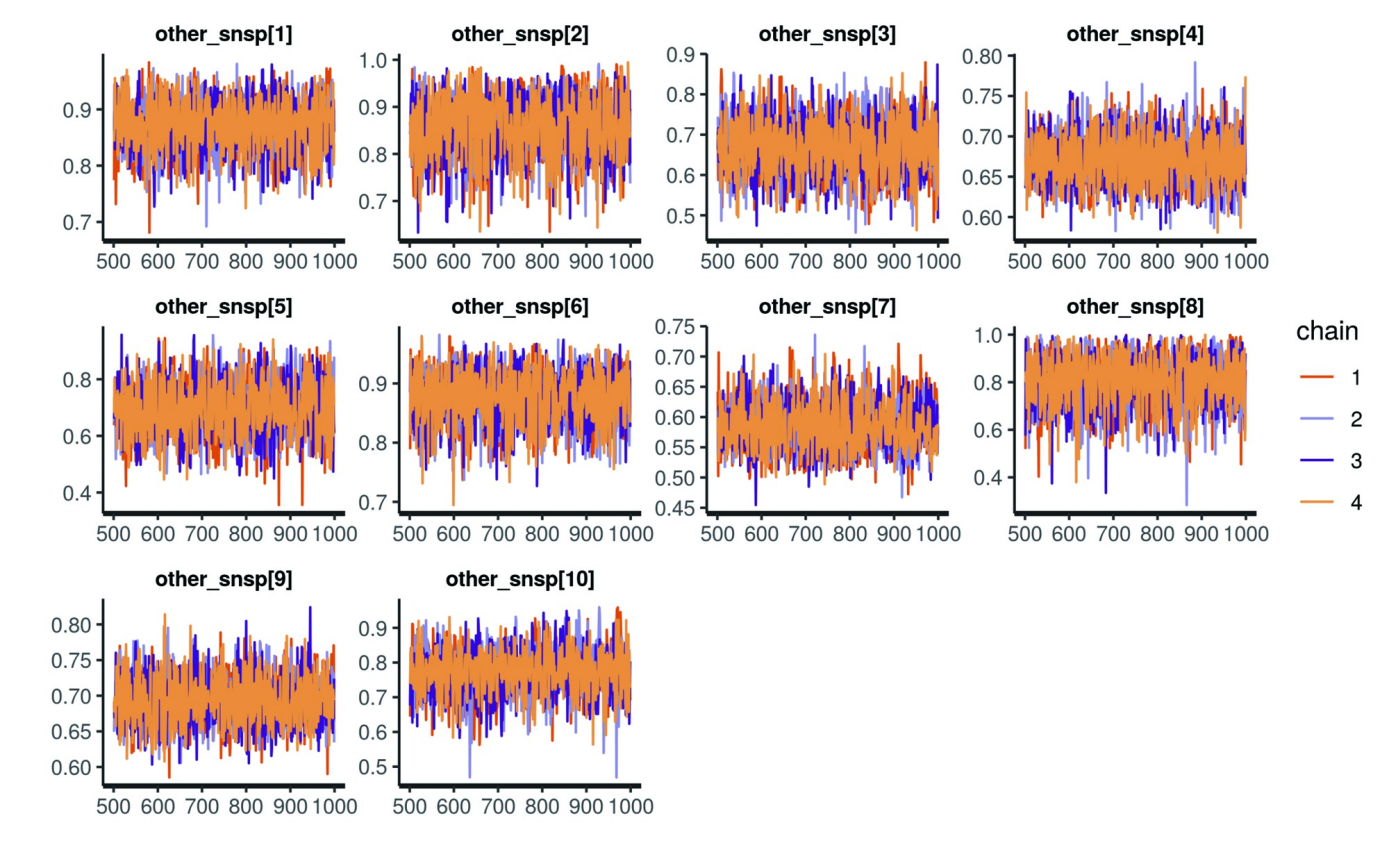
Example of Markov chain trace plot Other_snsp[max]” is a variable, which is an estimated probability.

The application also visualizes the Markov chain trace plot and shows MCMC method details after clicking the tab 'details about MCMC Method'. Users can download the Markov chain trace plot as a PNG file and download MCMC method details as a CSV file. Figure 3 is an example of a Markov chain trace plot. Table 2 presents examples of MCMC output values. “Other_snsp[max]” in Figure 3 and Table 2 is a variable and stands for estimated probability. We have attached the source codes for CAST-HSROC in Appendices 1 and 2, and the sample dataset is included in Table 3.

**Table 2:** Examples of Markov chain Monte Carlo output values Other_snsp[max]” is a variable, which is an estimated probability.

Statistics	Mean	se_mean	sd	X2.5.	X25.	X50.	X75.	X97.5.	n_eff	R^hat^
theta 1	0.422015	0.022683	0.423272	−0.3598	0.141642	0.402632	0.688426	1.309118	348.2215	1.008017
theta 2	−0.21382	0.021508	0.429972	−1.02926	−0.48258	−0.22375	0.054001	0.684675	399.6563	1.00566
theta 3	−0.72118	0.017955	0.351931	−1.41868	−0.93906	−0.71846	−0.48791	−0.04647	384.1763	1.007119
theta 4	0.417181	0.015053	0.28277	−0.11186	0.233797	0.408851	0.588236	1.028005	352.888	1.00815
theta 5	−0.07528	0.018739	0.434117	−0.91931	−0.35975	−0.08552	0.198351	0.816903	536.6791	1.00712
theta 6	−0.23934	0.023279	0.42331	−1.08943	−0.50421	−0.24436	0.028894	0.631096	330.6553	1.009076
theta 7	−0.16159	0.01206	0.253022	−0.65063	−0.32319	−0.15859	−0.0037	0.354414	440.1536	1.003977
theta 8	−0.13545	0.021521	0.4916	−1.13895	−0.44698	−0.12579	0.182429	0.83559	521.8081	1.005887
theta 9	0.528551	0.016516	0.302922	-0.03037	0.329454	0.515193	0.708076	1.201514	336.3792	1.010961
alpha 1	3.517859	0.014926	0.430751	2.777535	3.223045	3.490659	3.775899	4.438579	832.8332	1.003138
alpha 2	3.381596	0.014323	0.533385	2.421332	3.020807	3.360684	3.716789	4.458495	1386.712	1.001387
alpha 3	2.536311	0.017428	0.448603	1.757969	2.231187	2.514524	2.795491	3.507758	662.5856	1.005989
alpha 4	2.212084	0.012549	0.292161	1.74535	2.013196	2.178174	2.367714	2.959336	542.0352	1.008633
alpha 5	2.250259	0.017368	0.649863	0.927895	1.825288	2.246456	2.671653	3.528619	1399.994	1.00175
alpha 6	3.47381	0.012351	0.406631	2.734022	3.186354	3.467465	3.734397	4.321635	1083.895	0.999722
alpha 7	1.45843	0.010168	0.336215	0.798532	1.233116	1.460115	1.665975	2.148768	1093.405	1.004242
alpha 8	3.094948	0.023428	0.80843	1.693772	2.554757	3.026057	3.563595	4.90355	1190.692	1.000331
alpha 9	2.442103	0.015764	0.326303	1.923703	2.227697	2.406432	2.617395	3.166297	428.4748	1.013563
beta	0.138255	0.026523	0.46564	−0.76217	−0.16886	0.140307	0.436148	1.116725	308.2164	1.010835
theta_g	−0.01723	0.01817	0.367983	−0.70799	−0.26276	−0.02303	0.21596	0.741779	410.1497	1.005953
alpha_g	2.711888	0.013666	0.406919	1.953079	2.45671	2.68784	2.943395	3.601247	886.6327	1.007881
s_theta	0.561636	0.006513	0.214235	0.292345	0.421366	0.518942	0.64782	1.109912	1082.14	1.005545
s_alpha	1.001177	0.013187	0.392549	0.457162	0.732615	0.931189	1.162099	1.995619	886.1823	1.002389
pi_1_1	0.87702	0.000709	0.03117	0.811974	0.858137	0.878786	0.899042	0.931094	1931.171	0.999609
pi_1_2	0.788675	0.001011	0.050791	0.68458	0.754286	0.792143	0.825031	0.880404	2522.953	0.998626
pi_1_3	0.618206	0.001041	0.048402	0.523085	0.585153	0.619256	0.652007	0.709631	2162.461	1.001321
pi_1_4	0.798476	0.000466	0.024507	0.749127	0.782303	0.799114	0.815815	0.843993	2768.059	0.9999
pi_1_5	0.712132	0.001947	0.086738	0.529839	0.654836	0.721314	0.775227	0.858817	1984.67	1.000135
pi_1_6	0.79322	0.000788	0.036905	0.718296	0.768428	0.793702	0.819767	0.861836	2194.211	0.998921
pi_1_7	0.623212	0.001164	0.048021	0.524663	0.591136	0.625019	0.656947	0.714258	1701.797	1.000893
pi_1_8	0.77154	0.001866	0.088379	0.564559	0.718821	0.779356	0.833728	0.916607	2242.817	1.001374
pi_1_9	0.829797	0.000424	0.021416	0.786968	0.815486	0.830413	0.845034	0.869572	2546.903	0.999821
pi_0_1	0.201464	0.000714	0.038244	0.132333	0.174536	0.19988	0.226578	0.279772	2866.557	1.000124
pi_0_2	0.124981	0.000875	0.042253	0.052793	0.093978	0.120916	0.150621	0.216112	2334.247	1.001081
pi_0_3	0.114078	0.000668	0.031286	0.058818	0.091782	0.112551	0.134113	0.181282	2192.754	1.000392
pi_0_4	0.328672	0.000526	0.027017	0.277692	0.309389	0.329196	0.347223	0.380212	2641.595	0.999169
pi_0_5	0.232136	0.001733	0.078133	0.105514	0.175327	0.224163	0.279912	0.404675	2032.429	1.000315
pi_0_6	0.11501	0.000665	0.029716	0.062922	0.093565	0.113231	0.132972	0.179965	1997.549	0.999652
pi_0_7	0.28626	0.001008	0.044595	0.204523	0.254831	0.283762	0.315248	0.37985	1956.674	1.001227
pi_0_8	0.160014	0.001835	0.076612	0.039718	0.104698	0.149898	0.204152	0.336281	1743.322	1.001377
pi_0_9	0.327956	0.000577	0.027232	0.276256	0.309005	0.327447	0.346965	0.380014	2227.295	0.998933
sn 1	0.87702	0.000709	0.03117	0.811974	0.858137	0.878786	0.899042	0.931094	1931.171	0.999609
sn 2	0.788675	0.001011	0.050791	0.68458	0.754286	0.792143	0.825031	0.880404	2522.953	0.998626
sn 3	0.618206	0.001041	0.048402	0.523085	0.585153	0.619256	0.652007	0.709631	2162.461	1.001321
sn 4	0.798476	0.000466	0.024507	0.749127	0.782303	0.799114	0.815815	0.843993	2768.059	0.9999
sn 5	0.712132	0.001947	0.086738	0.529839	0.654836	0.721314	0.775227	0.858817	1984.67	1.000135
sn 6	0.79322	0.000788	0.036905	0.718296	0.768428	0.793702	0.819767	0.861836	2194.211	0.998921
sn 7	0.623212	0.001164	0.048021	0.524663	0.591136	0.625019	0.656947	0.714258	1701.797	1.000893
sn 8	0.77154	0.001866	0.088379	0.564559	0.718821	0.779356	0.833728	0.916607	2242.817	1.001374
sn 9	0.829797	0.000424	0.021416	0.786968	0.815486	0.830413	0.845034	0.869572	2546.903	0.999821
sp 1	0.798536	0.000714	0.038244	0.720228	0.773422	0.80012	0.825464	0.867667	2866.557	1.000124
sp 2	0.875019	0.000875	0.042253	0.783888	0.849379	0.879084	0.906022	0.947207	2334.247	1.001081
sp 3	0.885922	0.000668	0.031286	0.818718	0.865887	0.887449	0.908218	0.941182	2192.754	1.000392
sp 4	0.671328	0.000526	0.027017	0.619788	0.652777	0.670804	0.690611	0.722308	2641.595	0.999169
sp 5	0.767864	0.001733	0.078133	0.595325	0.720088	0.775837	0.824673	0.894486	2032.429	1.000315
sp 6	0.88499	0.000665	0.029716	0.820035	0.867028	0.886769	0.906435	0.937078	1997.549	0.999652
sp 7	0.71374	0.001008	0.044595	0.62015	0.684752	0.716238	0.745169	0.795477	1956.674	1.001227
sp 8	0.839986	0.001835	0.076612	0.663719	0.795848	0.850102	0.895302	0.960282	1743.322	1.001377
sp 9	0.672044	0.000577	0.027232	0.619986	0.653035	0.672553	0.690995	0.723744	2227.295	0.998933
other_snsp 1	0.873609	0.00103	0.046755	0.774879	0.84207	0.877268	0.908629	0.953542	2061.738	0.999358
other_snsp 2	0.860937	0.00148	0.064521	0.719973	0.819831	0.866332	0.909425	0.964793	1900.547	0.998982
other_snsp 3	0.671082	0.001594	0.070354	0.532346	0.621912	0.672281	0.720344	0.804813	1947.547	1.001663
other_snsp 4	0.670651	0.000563	0.031127	0.613433	0.649553	0.668955	0.690758	0.732259	3061.129	0.999284
other_snsp 5	0.689961	0.002347	0.097594	0.509735	0.619307	0.686606	0.759052	0.881747	1728.783	0.999266
other_snsp 6	0.876572	0.001162	0.046116	0.777165	0.846515	0.881552	0.911164	0.953098	1575.413	0.999213
other_snsp 7	0.583362	0.001009	0.039228	0.514229	0.554946	0.580981	0.608037	0.670708	1512.567	1.002244
other_snsp 8	0.80622	0.002518	0.111694	0.564753	0.73158	0.815108	0.892495	0.982608	1968.008	0.998871
other_snsp 9	0.694598	0.000696	0.03445	0.630637	0.669715	0.693987	0.718687	0.763395	2447.01	0.998363
other_snsp 10	0.771449	0.001708	0.064678	0.636526	0.732622	0.771651	0.812827	0.900959	1433.766	1.000926
lp__	−1116.79	0.146802	3.748451	−1125.38	−1119	−1116.33	−1114.17	−1110.61	651.9841	1.004291

**Table 3:** Sample data to input Sample data provided in the application, for users to download.

Study_name	TP	FN	FP	TN
1995-Andrew	90	10	20	80
1998-Philips	40	10	5	45
2003-Lee	60	40	10	90
2008-Yamada	200	50	100	200
2013-Lewis	10	5	5	15
2014-Williams	80	20	10	90
2015-Wang	60	40	30	70
2016-Cooper	8	2	1	9
2018-Dunphy	250	50	100	200

Applying calculated data to the SoF table

**Figure 4:**
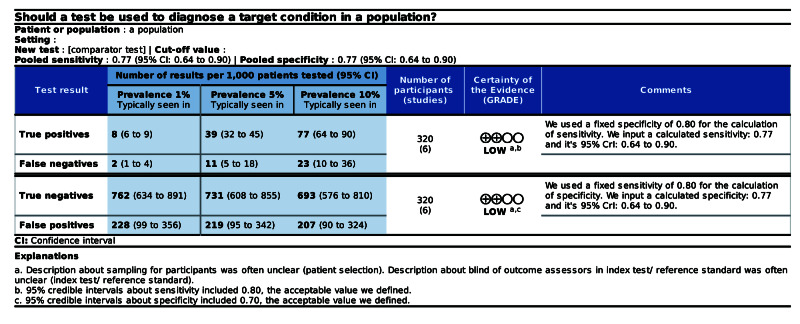
Example of the SoF table for meta-analyses of DTA SoF: summary of findings, DTA: diagnostic test accuracy, CrI: credible interval.

Users can input estimated probability and its 95% Crl to the SoF table. We present an example of an SoF table for DTA when utilizing the calculator (Figure 4).

## Results

Users should check whether convergence is reached before interpreting the results. There are two ways of achieving this: (1) checking whether the R^hat^ of other_snsp[max] is 1.1 or less in the MCMC method details, and (2) checking the Markov chain trace plot for other_snsp[max].

CAST-HSROC helps to easily calculate the point estimate of either sensitivity or specificity and its 95% CrI for a fixed specificity or sensitivity value in the HSROC model of DTA study, whereas existing GUI software such as Review Manager and MetaDTA cannot calculate the statistics (Table 4). Moreover, CAST-HSROC is a GUI software, in which researchers can input data more easily than a CUI software such as SAS and R (Table 4).

**Table 4:** HSROC-bivariate meta-analysis comparison between the software programs GUI: graphical user interface, CUI: character user interface, SROC: summary receiver operating characteristic, HSROC: hierarchical summary receiver operating characteristic, SAS: statistical analysis system, CAST-HSROC: calculator for the summary points from the HSROC model. ^a^The software can calculate the synthesized value of sensitivity or specificity when one of them is fixed.

Software	Review Manager	MetaDTA	SAS	R	CAST-HSROC
Price	Free	Free	Free in SAS University Edition	Free	Free
GUI/CUI	GUI	GUI	CUI	CUI	GUI
Summary points in bivariate meta-analysis	Yes (exported data from MetaDTA)	Yes	Yes	Yes	No
SROC in the HSROC model	Yes (exported data from MetaDTA)	Yes	Yes	Yes	No
Synthesized value of sensitivity or specificity in the HSROC model	No	No	Yes^a^	Yes^a^	Yes^a^

## Discussion

CAST-HSROC can help to create an SoF table for DTA when systematic reviewers or guideline developers estimate the certainty of evidence on the HSROC model. Moreover, the point estimate of either sensitivity or specificity and its 95% CrI for a fixed specificity or sensitivity value might be helpful for researchers aiming to calculate the values of sensitivity or specificity. SAS and R can also implement HSROC models and CAST-HSROC. These software products have CUIs, whereas CAST-HSROC has a GUI [9,12].

The software has some limitations. First, researchers who did not receive adequate statistical education might misunderstand the results of the HSROC model owing to cursory comprehension of the model or the result of qualitative synthesis in DTA. Therefore, we recommend that CAST-HSROC users read and understand the Cochrane Handbook and the GRADE working group’s guidelines for estimating the certainty of the evidence for DTA [2-4]. Assumptions that must be met at a minimum are that the user can use estimated statistics only when the MCMC converges. We also recommend the involvement of statisticians, if possible. Second, the setting of MCMC is fixed [6]. Third, the point estimate of either sensitivity or specificity and its 95% CrI for a fixed specificity or sensitivity value derived from this software may be complementary to the HSROC model. The HSROC curve itself is the main output of the HSROC model. MetaDTA can obtain the curve, although CAST-HSROC does not create the curve [13].

## Conclusions

CAST-HSROC can easily calculate the point estimate of either sensitivity or specificity and its 95% CrI for a fixed specificity or sensitivity value on the HSROC model when systematic reviewers or guideline developers estimate the certainty of evidence on the HSROC model. The point estimate of either sensitivity or specificity and its 95% CrI for a fixed specificity or sensitivity value, derived from CAST-HSROC, may be complementary to the HSROC model. CAST-HSROC is useful because existing GUI software products, such as Review Manager and MetaDTA, cannot calculate the point estimate of either sensitivity or specificity and its 95% CrI for a fixed specificity or sensitivity value on the HSROC model.

## Appendices

Appendix 1

Title of Data

Archive of source code of the current version of CAST-HSROC

Description of Data

The code used to construct the application

library(shiny) library(shinythemes) library(rstan) library(tidyverse) library(ggplot2) library(HDInterval) lmcss <- " #plot-container1 { position: absolute; left: 50%; top: 40%; z-index: -1; } #loading-spinner1 { position: absolute; left: 50%; top: 50%; z-index: -1; margin-top: -33px; /* half of the spinner's height */ margin-left: -33px; /* half of the spinner's width */ } #plot.recalculating { z-index: -2; } #loadmessage1 { position: absolute; top: 50%; left: 10%; width: 80%; padding: 5px 0px 5px 0px; text-align: center; font-size:130%; font-style:italic; color: #708090; background-color:white; z-index: -1; } " df = read.csv("sampledata_wide.csv") ui <- fluidPage( fluidRow( headerPanel(h2("CAST-HSROC: CAlculator for the Summary poinT from HSROC model"), windowTitle = "CAST-HSROC: Calculator for the Summary point from HSROC Model") ), fluidRow( navbarPage(theme = shinytheme("flatly"), "Menu", tabPanel("Introduction", style = "position:absolute; marginleft: 80px; margin-right: 100px", h3("Estimating the Certainty of Evidence for HSROC Model in the Meta-analysis of Diagnostic Test Accuracy", align = "center"), br(), p("The Grading of Recommendations Assessment, Development and Evaluation (GRADE) Working Group have published a new guideline regarding estimating the certainty of evidence for the meta-analysis of diagnostic test accuracy (DTA) [1]. However, no concensus has been achieved on how to estimate the certainty of evidence for ", span("hierarchical summary receiver operating characteristic (HSROC) model.", style = "color:lightseagreen"), style = "font-size:19px"), p("HSROC model is a statistical model based on latent scale logistic regression. It considers variability both within and between studies (for example, different thresholds used in primary studies) [2]. HSROC cannot estimate a summary point of sensitivity and specificity. Instead, we can present", strong("the estimate of specificity and its 95% credible interval (CrI), if a fixed value of sensitity is given, or vice versa,", style = "color:steelblue"), " to demonstrate the changes in sensitivity and specificity along the curve.", style = "font-size:19px"), p("We think this result should be included in the Summary of Finding (SoF) table for a meta-analysis of DTA using HSROC model. However, in order to obtain these values, difficult calculation based on log diagnostic odds ratio (log DOR) is needed [2]. Therefore, we develop this calculator for users to easily estimate it. We also present ", strong("an example of the SoF table for meta-analyses of DTA", style = "color:steelblue"), " in below: ", style = "font-size:19px"), div(img(src = "sof_table.png", height = "200%", width = "80%"), align = "center"), hr(), p("[1] Schunemann HJ, Mustafa RA, Brozek J, Steingart KR, Leeflang M, Murad MH, et al. GRADE guidelines: 21 part 2. Inconsistency, Imprecision, publication bias and other domains for rating the certainty of evidence for test accuracy and presenting it in evidence profiles and summary of findings tables. J Clin Epidemiol 2020.", style = "font-size: 15px; color: grey"), p("[2] Deeks JJ, Bossuyt PM, Gatsonis C (editors). Cochrane Handbook for Systematic Reviews of Diagnostic Test Accuracy Version 1.0. Available from: http:// srdta.cochrane.org/.; 2010 [accessed March 1 2020].", style = "font-size: 15px; color: grey") ), tabPanel("Upload Data", sidebarLayout( sidebarPanel(width = 3, fileInput("file", label = "Please select the file", multiple = FALSE, accept = c("text/csv","text/comma-separatedvalues, text/plain",".csv") ), tags$hr(style = "border-color: lightgray;"), strong("File Options", style = "font-size: 16px"), br(), checkboxInput("header", label = "First row as column headings", value = TRUE), br(), radioButtons("sep", label = "File Delimiter/ Separator", choices = c(Comma = ",", Semicolon = ";", Tab = "\t", Space = " "), selected = ","), br(), radioButtons("display", label = "Display the full dataset or the first 10 rows", choices = c(All = "all", Head = "head"), selected = "all"), tags$hr(style="border-color: lightgray;"), strong("Download the example dataset", style = "fontsize: 16px"), br(), br(), downloadButton("dlexample", label = "Download the example") ), mainPanel( tabsetPanel( tabPanel("Data Format Requirement", br(), h4("Please upload your file first."), br(), p("(1) The file should be in the formats that use delimiter-separated values (DSV), meaning that to store two-dimensional arrays of data by separating the values in each row with specific delimiter characters. The supported delimiters are: comma (,), semicolon (;), tab (\t), and space ( ). Please make sure to select the corresponsing file delimiter in the left panel. ", strong("We recommend to upload a comma-separated values (CSV) file!", style = "color:steelblue"), style = "font-size:17px"), br(), p("(2) The dataset should have 5 columns.", style = "font-size:17px"), p(strong("Column 1"), "should be named as", strong(" 'study_name' ", style = "color:steelblue"), ", referring to the study ID, which can be can be numerics or characters. Each study contains fourfold (2x2) table information.", style = "font-size:17px"), p(strong("Column 2"), "should be named as", strong(" 'TP' ", style = "color:steelblue"), ", the number of true positive patients (disease + positive result).", style = "font-size:17px"), p(strong("Column 3"), "should be named as", strong(" 'FN' ", style = "color:steelblue"), ", the number of false negative patients (disease + negative result).", style = "font-size:17px"), p(strong("Column 4"), "should be named as", strong(" 'FP' ", style = "color:steelblue"), ", the number of false positive patients (no disease + positive result).", style = "font-size:17px"), p(strong("Column 5"), "should be named as", strong(" 'TN' ", style = "color:steelblue"), ", the number of true negative patients (no disease + negative result).", style = "font-size:17px"), br(), p("(3) If you upload the dataset successfully, you can visualize your data by clicking the tab", strong(" 'Data Confirmation' ", style = "color:steelblue"), style = "font-size:17px"), br(), p("(4) The example dataset looks like this in below. You can also download it. ", style = "font-size:17px"), div(tableOutput("showexample"), style = "marginleft: 150px"), br(), br() ), tabPanel("Data Confirmation", br(), tableOutput("dataconfirm") ) ) ) ) ), tabPanel("Results", sidebarLayout( sidebarPanel(width = 3, strong("Input the parameter", style = "font-size: 18px"), br(), br(), radioButtons("parametername", label = "Choose the class of input parameter", choices = c("Sensitivity", "Specificity"), selected = "Sensitivity"), textInput("parameter", label = "Input the parameter value", placeholder = "Enter your parameter value"), tags$hr(style="border-color: lightgray;"), strong("Note: ", style = "color:gray"), p("*The ", span("sensitivity or specificity parameter", style = "color:steelblue;font-style:italic"), " can be selected based on the clinical experience or previous studies. For example, it can be the average value of the studies included in the metaanalysis.", style = "color:gray"), withMathJax(), p("**The HSROC model takes the form [1]: ", span("$$logit(sensitivity_i) = (\\theta_i + 0.5\ \alpha_i)exp(-0.5\\beta)$$ $$logit(specificity_i) = 1 - (\\theta_i - 0.5\ \alpha_i)exp(0.5\\beta)$$", style = "font-size:13px"), "\\(i\\) refers to the \\(i\\)th study. If we set sensitivity or specificity to 0.5, the left side of the above equation becomes 0. Since exp is constantly positive, \\(\\beta\\) can be any value, making it an indeterminate equation. Therefore, if you input a sensitivity or specificity value as 0.5, you may get an error message.", style = "color:gray"), p("***We provide the details about the MCMC methods. You should check if convergence is reached before interpreting the result. There are two ways: (1) to check if the Rhat of other_snsp[max] is 1.1 or less; (2) to check Markov Chain Trace Plot for other_snsp[max].", style = "color:gray"), p("****The model used in this calculator was built in RStan. The code can be found at:", span("https://github.com/y-luo06/HSROC_shiny.", style = "color:lightseagreen;font-style:italic"), style = "color:gray"), tags$hr(style="border-color: lightgray;"), p("[1] Macaskill P, Gatsonis C, Deeks J, Harbord R, Takwoingi Y. Cochrane handbook for systematic reviews of diagnostic test accuracy. The Cochrane Collaboration 2010.", style = "font-size: 14px; color: grey") ), mainPanel( tabsetPanel( tabPanel("Results", style = "margin-left:20px;", fluidRow( h4("Estimation"), br(), uiOutput("estimation"), br(), br(), tags$hr(style="border-color: lightgray;"), ), fluidRow( h4("Probability Density Plot"), br(), #Add a loading message tags$head(tags$style(HTML(lmcss))), conditionalPanel(condition = "$ ('html').hasClass('shiny-busy')", tags$div(id = "plotcontainer1", tags$img(src = "spinner.gif", id = "loading-spinner1")), tags$div("Please wait a moment for the analysis to finish.",id="loadmessage1")), div(plotOutput("densityplot", width = "600px", height = "500px"), align = "center"), br(), p("You can download this probability density plot here.", style = "font-size:17px"), downloadButton("dldensityplot", label = "Download the plot"), br(), br() ) ), tabPanel("Details about MCMC Method", style = "margin-left:20px;", fluidRow( h4("Markov Chain Trace Plot"), br(), #Add a loading message tags$head(tags$style(HTML(lmcss))), conditionalPanel(condition = "$ ('html').hasClass('shiny-busy')", tags$div(id = "plotcontainer1", tags$img(src = "spinner.gif", id = "loading-spinner1")), tags$div("Please wait a moment for the analysis to finish.",id="loadmessage1")), div(plotOutput("traceplot", width = "80%", height = "500px"), align = "center"), br(), p("You can download this trace plot here.", style = "font-size:17px"), downloadButton("dltraceplot", label = "Download the plot"), br(), hr() ), fluidRow( h4("MCMC Method Details"), verbatimTextOutput("mcmcfit"), br(), p("You can download it in the form of csv here.", style = "font-size:17px"), downloadButton("dlfit", label = "Download the file"), br() ) ) ) ) ) ) ) ), fluidRow(column(2,div(style = "height:1000px;backgroundcolor: rgba(0,0,0,0);"))), fluidRow( fillRow(div(style = "height:110px;backgroundcolor: rgba(229,232,235,1);")), br(), column(1), column(1, br(), img(src = "ccby.png", height = 30, width = 80, align = "center")), column(10, strong("Please cite:", style = "color:rgba(21,45,70,1)"), p("Banno M, Tsujimoto Y, Luo Y, Miyakoshi C, Kataoka Y. Estimating the certainty of evidence in Grading of Recommendations Assessment, Development and Evaluation for test accuracy.", em(" (In submission)"), style = "font-size: 14px; color:rgba(21,45,70,1)") ) ) ) server <- function(input, output, session) { input_file <- reactive({ read.csv(input$file$datapath, header = input$header, sep = input$sep) }) file_reshape <- reactive({ df.tmp <- read.csv(input$file$datapath, header = input$header, sep = input$sep) df.tmp$study <- seq.int(1, length(unique(df.tmp$study_name))) names(df.tmp)[names(df.tmp) == "TP"] <- "np.1" names(df.tmp)[names(df.tmp) == "FN"] <- "nn.1" names(df.tmp)[names(df.tmp) == "FP"] <- "np.0" names(df.tmp)[names(df.tmp) == "TN"] <- "nn.0" reshape(data = df.tmp, idvar = "study_name", direction = "long", varying = c(2:5), timevar = "status", sep = ".") }) output$dlexample <- downloadHandler( filename = "example_dataset.csv", content = function(file){ file.copy("sampledata_wide.csv", file) } ) output$showexample <- renderTable(bordered = TRUE, striped = TRUE, { return(head(df, 6)) }) output$dataconfirm <- renderTable(bordered = TRUE, striped = TRUE, { req(input$file) if (input$display == "head") { return(head(input_file(), 10)) } else { return(input_file()) } }) fit <- reactive({ parameter <- as.numeric(input$parameter) req(input$file, input$parameter, parameter > 0 & parameter <1 & parameter != 0.5) datalist <- list( N = nrow(file_reshape()), I = length(unique(file_reshape()$study)), study = file_reshape()$study, status = file_reshape()$status, np = file_reshape()$np, nn = file_reshape()$nn, one_snsp = parameter ) model <- stan_model("HSROC.stan") set.seed(1234) fit <- sampling(model, data = datalist, iter = 1000, warmup = 500, chains = 4, thin = 1, cores = 4) }) other_snsp <- reactive({ other_snsp <- rstan::extract(fit())$other_snsp list = colnames(other_snsp) <- c(as.character(input_file()$study_name), "all") other_snsp <- as.data.frame(other_snsp) %>% tidyr::gather(key = "study_name",value = "prob") }) other_snsp_global <- reactive({ filter(other_snsp(), study_name == "all") }) densityplot <- reactive({ ggplot() + geom_density(data = other_snsp_global(), aes(x=prob), fill = "red", alpha = 0.2) + geom_density(data = other_snsp(), aes(x=prob, color = study_name)) }) traceplot <- reactive({ stan_trace(fit(), pars = "other_snsp") }) output$estimation <- renderUI({ parameter <- as.numeric(input$parameter) validate( need(input$file, "Please make sure to upload a dataset with required format."), need(parameter >= 0 & parameter <= 1, "Please make sure to input a correct parameter value between 0 to 1.") ) if (parameter == 0.5) { withMathJax( helpText("Please input a parameter value other than 0.5.", "This is because HSROC model takes the form [1]: $$logit(sensitivity_i) = (\\theta_i + 0.5\ \alpha_i)exp(-0.5\\beta)$$ $$logit(specificity_i) = 1 - (\\theta_i - 0.5\ \alpha_i)exp(0.5\\beta)$$ \\(i\\) refers to the \\(i\\)th study. If we set sensitivity or specificity to 0.5, the left side of the above equation becomes 0. Since exp is constantly positive, \\(\\beta\\) can be any value, making it an indeterminate equation. Therefore, please input a value other than 0.5.")) } else { if (input$parametername == "Sensitivity") { paste("The estimated specificity is ", round(mean(other_snsp_global()$prob), digits = 3), ", with a 95% credible interval from ", format(round(hdi(other_snsp_global()$prob)[[1]], digits = 3), nsmall = 3), " to ", format(round(hdi(other_snsp_global()$prob)[[2]], digits = 3), nsmall = 3), ".") } else { paste("The estimated sensitivity is ", round(mean(other_snsp_global()$prob), digits = 3), ", with a 95% credible interval from ", format(round(hdi(other_snsp_global()$prob)[[1]], digits = 3), nsmall = 3), " to ", format(round(hdi(other_snsp_global()$prob)[[2]], digits = 3), nsmall = 3), ".") } } }) output$densityplot <- renderPlot({ req(input$file, input$parameter) densityplot() }) output$dldensityplot <- downloadHandler( filename = "probability_density_plot.png", content = function(file){ ggsave(file, plot = densityplot(), width = 6, height = 5, device = "png") } ) output$traceplot <- renderPlot({ req(input$file, input$parameter) traceplot() }) output$dltraceplot <- downloadHandler( filename = "trace_plot.png", content = function(file){ ggsave(file, plot = traceplot(), width = 8, height = 5, device = "png") } ) output$mcmcfit <- renderPrint({ print(fit()) }) output$dlfit <- downloadHandler( filename = "fit_summary.csv", content = function(file){ write.table(data.frame(summary(fit())$summary), file, sep=',', row.names = TRUE, col.names = NA) } ) } shinyApp(ui = ui, server = server)

Appendix 2

Title of Data

Archive of source code for the current version of CAST-HSROC

Description of Data

The model used to calculate the value of interest based on the HSROC model

data{ int N; //number of row of datasheet int I; //number of studies included int study[N]; //study ID int status[N]; //dummy variable for patient status (1 for pts w/ dis,0 for pts w/o dis) int np[N]; //number of patients with positive test result in each status int nn[N]; //number of patients with negative test result in each status real<lower=0,upper=1> one_snsp; //fix one of sn/sp } parameters{ vector[I] theta; //theta for each study vector[I] alpha; //alpha for each study real beta; //common scale parameter real theta_g; //global mean of theta real alpha_g; //global mean of alpha real<lower=0> s_theta; //square of between study variance for theta real<lower=0> s_alpha; //square of between study variance for alpha } transformed parameters{ vector<lower=0,upper=1>[I] pi_1; //prob of having positive result among pts w/ dis vector<lower=0,upper=1>[I] pi_0; //prob of having positive result among pts w/o dis vector<lower=0,upper=1>[I] sn; //sensitivity of each study(=pi_1) vector<lower=0,upper=1>[I] sp; //specificitiy of each study(=-pi_0) vector<lower=0,upper=1>[I+1] other_snsp; pi_1 = inv_logit((theta + 0.5*alpha)*exp(-0.5*beta)); pi_0 = inv_logit((theta - 0.5*alpha)*exp(0.5*beta)); sn = pi_1; sp = 1-pi_0; for(i in 1:I){ other_snsp[i] = inv_logit((0.25*(alpha[i]^2) - theta[i]^2)/ log(one_snsp/(1-one_snsp))); } other_snsp[I+1] = inv_logit((0.25*(alpha_g^2) - theta_g^2)/log(one_snsp/ (1-one_snsp))); } model{ for(n in 1:N){ if(status[n]==1){ np[n] ~ binomial(np[n]+nn[n],pi_1[study[n]]); //Level I model for true positive }else{ np[n] ~ binomial(np[n]+nn[n],pi_0[study[n]]); // Level I modek for false positive } } theta ~ normal(theta_g,s_theta); //Level II alpha ~ normal(alpha_g,s_alpha); //Level II }
